# Hepatitis B–Related Hepatic Flare During Immune Reconstitution Syndrome After Antiretroviral Treatment Initiation in an HBV Surface Antigen–Positive Patient With HIV: Viroimmunological and Histological Characterization

**DOI:** 10.1093/ofid/ofac451

**Published:** 2022-08-29

**Authors:** Marco Iannetta, Angela M A Crea, Andrea Di Lorenzo, Laura Campogiani, Elisabetta Teti, Vincenzo Malagnino, Mirko Compagno, Luigi Coppola, Lorenzo Piermatteo, Giampiero Palmieri, Carolina Cimino, Romina Salpini, Maria A Zingaropoli, Maria R Ciardi, Claudio M Mastroianni, Saverio G Parisi, Valentina Svicher, Massimo Andreoni, Loredana Sarmati

**Affiliations:** Department of System Medicine, Tor Vergata University, Rome, Italy; Department of System Medicine, Tor Vergata University, Rome, Italy; Department of System Medicine, Tor Vergata University, Rome, Italy; Department of System Medicine, Tor Vergata University, Rome, Italy; Department of System Medicine, Tor Vergata University, Rome, Italy; Department of System Medicine, Tor Vergata University, Rome, Italy; Department of System Medicine, Tor Vergata University, Rome, Italy; Department of System Medicine, Tor Vergata University, Rome, Italy; Department of Experimental Medicine, Tor Vergata University, Rome, Italy; Department of Biomedicine and Prevention, Tor Vergata University, Rome, Italy; Department of Biomedicine and Prevention, Tor Vergata University, Rome, Italy; Department of Experimental Medicine, Tor Vergata University, Rome, Italy; Department of Public Health and Infectious Diseases, Sapienza University, Rome, Italy; Department of Public Health and Infectious Diseases, Sapienza University, Rome, Italy; Department of Public Health and Infectious Diseases, Sapienza University, Rome, Italy; Department of Molecular Medicine, University of Padova, Padua, Italy; Department of Experimental Medicine, Tor Vergata University, Rome, Italy; Department of Biology, Tor Vergata University, Rome, Italy; Department of System Medicine, Tor Vergata University, Rome, Italy; Department of System Medicine, Tor Vergata University, Rome, Italy

**Keywords:** HBV, AIDS, IRIS, biopsy, cccDNA, immune activation, pgRNA

## Abstract

HIV and hepatitis B virus (HBV) coinfection is relatively common. Initiation of antiretroviral therapy (ART) in people with HIV (PWH) causes a progressive restoration of cell-mediated immune functions. In the presence of overt or occult coinfections, immune restoration might lead to immune reconstitution inflammatory syndrome (IRIS). Here, we describe the clinical, immunological, virological, and histological characterization of a case of HBV-related IRIS hepatitis in a PWH after ART initiation. A liver biopsy was performed during HBV-related IRIS hepatic flare, and liver samples were analyzed through immunohistochemistry and molecular techniques, with the assessment of intrahepatic HBV-DNA, covalently closed circular DNA, and HBV pregenomic RNA through a droplet digital polymerase chain reaction system. Immune activation and senescence were also longitudinally assessed. In this clinical case, the hepatic flare occurred 6 weeks after ART initiation with a therapeutic regimen including tenofovir alafenamide (TAF) and emtricitabine (FTC). The episode was self-limiting, characterized by hyperactivation of peripheral blood CD4+ and CD8+ T-lymphocytes, and resolved without ART discontinuation, leading to the achievement of HBsAg seroconversion (HBsAg-/HBsAb+) and HBV-DNA plasma undetectability. Notably, hyperactivation of the immune system plays a pivotal role in promoting the control of HBV replication, thus triggering the achievement of HBsAg seroconversion during treatment with TAF/FTC.

Viral hepatitis is still a major public health issue. For hepatitis B virus (HBV), the introduction of vaccination and effective antiviral therapies for chronic suppression have substantially reduced the spread of HBV-related hepatitis and its complications [1, 2]. In Italy, epidemiological studies investigating HBV surface antigen (HBsAg) seroprevalence have shown a progressive decrease over the last 4 decades, with a current prevalence of nearly 1% in the general population and a heterogeneous geographical distribution characterized by an increasing north–south gradient [2, 3].

HIV and HBV infection share the same transmission routes, and coinfection is relatively common. It has been estimated that worldwide ∼12% of people with HIV (PWH) are coinfected with HBV [4]. An Italian survey performed in Tuscany in 2016 reported a prevalence of 4.1% for HBV coinfection in PWH and 0.3% for HBV/HCV coinfection. Some studies have shown a negative influence of HBV coinfection on response to HIV antiretroviral therapy (ART) and immunological recovery in PWH [5, 6]. HIV/HBV coinfection also leads to a more rapid progression of hepatic fibrosis and chronic complications, including cirrhosis, hepatocellular carcinoma, and hepatic failure requiring liver transplantation [7]. Initiation of ART in PWH causes a decline in HIV viral load with progressive restoration of CD4+ T-cell counts and cell-mediated immune functions. In the presence of overt or occult coinfections, immune restoration might lead to immune reconstitution inflammatory syndrome (IRIS), characterized by worsening clinical manifestation of the preexisting infection. In HIV/HBV patients, an enhanced immune response to HBV might cause the appearance or exacerbation of hepatitis, with increased liver function test values (hepatic flare) [8–10]. Hypertransaminasemia can also be a consequence of ART toxicity; distinguishing between immune reconstitution inflammatory syndrome (IRIS) and drug liver toxicity is essential for optimizing the clinical management of PWH with HBV coinfection at ART initiation.

Here, we describe the clinical, immunological, virological, and histological characterization of a case of HBV-related IRIS hepatitis in a PWH after ART initiation.

## METHODS

Liver enzymes (aspartate transaminase [AST], alanine transaminase [ALT]), white blood cell count, CD4+ and CD8+ T-cell counts, HBV-DNA, and HIV-RNA were routinely assessed in the central laboratory of Tor Vergata University Hospital.

Histological examination after hematoxylin and eosin staining and core and surface HBV antigen immunohistochemical staining was performed on a liver biopsy, following standard procedures.

Intrahepatic HBV-DNA (ihHBV-DNA), covalently closed circular DNA (cccDNA), and HBV pregenomic RNA (pgRNA) were assessed with droplet digital polymerase chain reaction (ddPCR) on a liver biopsy sample as previously shown [11, 12]. The HBV genotype was assessed by population-based sequencing of the reverse transcriptase (RT)/HBsAg genomic region [13] followed by phylogenetic analysis.

The T-cell phenotype was assessed with multiparametric flow cytometry after surface staining of fresh heparin whole-blood samples, following a lyse-and-wash protocol, as previously shown [14]. The following fluorochrome-conjugated antibodies were used for surface staining: CD3 Pacific Blue (clone HIT3a), CD4 Allophycocyanin (APC)-Cyanine (Cy)7 (clone RPA-T4), CD8 Phycoerythrin (PE)-Cy7 (clone SK1), CD38 APC (clone HIT2), CD57 PE (clone HCD57), CD28 Fluorescein-5-Isothiocyanate (FITC; clone CD28.2), and HLA-DR Peridinin-Chlorophyll-Protein (PerCP)-Cy5.5 (clone L243), purchased from BioLegend (San Diego, CA, USA).

## RESULTS

### Case Presentation

**Figure 1.**
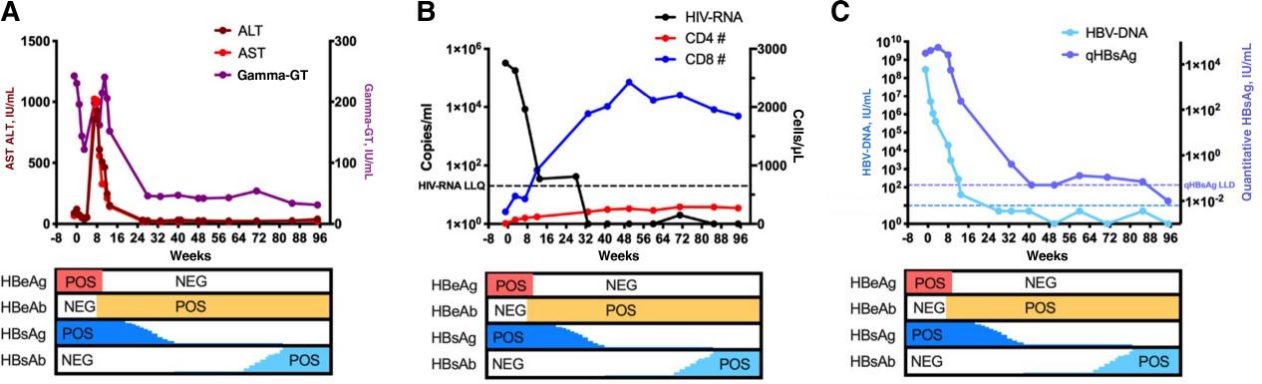
Description of the liver enzymes, HIV and HBV viral parameters, and T-lymphocyte absolute counts. Liver enzyme trend (A), HIV-RNA viral load and CD4+ and CD8+ T-lymphocyte absolute counts (B), HBV-DNA viral load and quantitative HBsAg trend (C) over time. Week 0 represents initiation of antiretroviral therapy. HBV-DNA was assessed using the COBAS AmpliPrep/COBAS TaqMan system with a lower limit of quantification of 10 IU/mL; HIV-RNA was assessed using the COBAS AmpliPrep/COBAS TaqMan system with a lower limit of quantification of 10 IU/mL. Quantitative HBsAg was assessed with a chemiluminescent assay with a lower limit of detection of 0.05 IU/mL. Abbreviations: ALT, alanine aminotransferase; AST, aspartate aminotransferase; CD4#, CD4+ T-lymphocyte absolute count; CD8#, CD8+ T-lymphocyte absolute count; gamma-GT, gamma glutamyl transferase; HBeAb, anti–hepatitis B e antibody; HBeAg, hepatitis B e antigen; HBsAg, hepatitis B s antigen; HBsAb, anti–hepatitis B s antibody; HBV, hepatitis B virus; LLD, lower limit of detection; LLQ, lower limit of quantification; NEG, negative; POS, positive; qHBsAg, quantitative hepatitis B s antigen.

A 51-year-old Caucasian man was hospitalized due to pancytopenia and a cancerous lesion of the second finger of the left hand, which was histologically characterized as a squamous cell carcinoma. During the pancytopenia workup, HIV-1 (subtype B) infection was diagnosed, and HBV (genotype A) coinfection was identified. The patient had never received HBV vaccination. The CD4+ T-cell count was 9 cells/µL (3%), with a CD4/CD8 ratio of 0.04. HIV-RNA was 3.3 × 10^5^ copies/mL, and HBV-DNA was 2.94 × 10^8^ IU/mL. HBV surface antigen (HBsAg) and HBV e antigen (HBeAg) were both positive. The quantitative HBsAg titer was 30 848 IU/mL. Antibodies against HBsAg (HBsAb) and HBeAg (HBeAb) were negative (Figure 1 and Table 1).

**Table 1.** White Blood Cells, Liver Enzymes, and Viroimmunological Parameters in Plasma and Liver Samples

Parameter	Pre-ART	T0	T4w	T8w	T12w	T25w	T40w	T85w	T96w
WBC, cells/µL	1620	1160	2590	2270	4450	8620	5890	7080	6310
CD4, cells/µL	9	NA	71	100	122	NA	245	288	270
CD8, cells/µL	208	NA	476	428	923	NA	2011	1955	1845
CD4/CD8 ratio	0.04	NA	0.15	0.23	0.13	NA	0.12	0.15	0.15
ALT, U/L	67	90	55	1010	211	24	24	26	41
AST, U/L	89	121	53	927	257	32	32	26	40
HBV-DNA, IU/mL	294 × 10^6^	NA	4.11 × 10^5^	20 × 10^3^	277	TD <10	TD <10	TD <10	TND
qHBsAg, IU/mL	30 848	…	55 460	26 221	240	0.41	0.05	0.07	0.00
HIV-RNA, copies/mL	330 × 10^3^	NA	180 × 10^3^	8.4 × 10^3^	34.9	41.7	TND	TND	TND
ihHBV-DNA,^a^ copies/1000 cells	NA	NA	NA	324	NA	NA	NA	NA	NA
HBV pgRNA,^a^ copies/1000 cells	NA	NA	NA	3.3 × 10^6^	NA	NA	NA	NA	NA
HBV cccDNA,^a^ copies/1000 cells	NA	NA	NA	141	NA	NA	NA	NA	NA

T0 represents the initiation of ART.

Abbreviations: ALT, alanine aminotransferase; ART, antiretroviral treatment; AST, aspartate aminotransferase; HBV, hepatitis B virus; HBV cccDNA, HBV covalently closed circular DNA; HBV pgRNA, HBV pregenomic RNA; ihHBV-DNA, intrahepatic HBV-DNA; NA, not available; TD, target detected; TND, target not detected; WBC, white blood cells.

a ih-HBV-DNA, HBV pgRNA, and cccDNA were assessed in the liver biopsy.

Active and latent tuberculosis infections and cryptococcal infections were excluded. Human cytomegalovirus (CMV)-DNA was detectable in the blood (2188 IU/mL), and CMV chorioretinitis was diagnosed (cotton wool spots in the retina). Treatment with 2 weeks of intravenous ganciclovir followed by 2 weeks of oral valganciclovir was started, and CMV-DNA viremia progressively decreased until becoming persistently undetectable after 1 month of therapy.

ART was promptly started with bictegravir/emtricitabine/tenofovir alafenamide (BIC/FTC/TAF), which was also active for HBV infection. Pre-ART liver enzymes were slightly altered, with ALT and AST of 67 and 89 U/L, respectively; ALT and AST values improved during the first month of treatment (43 and 39 U/L, respectively). Six to 8 weeks after ART initiation during outpatient follow-up visits, despite a decrease in HIV-RNA and HBV-DNA (Figure 1 and Table 1), the patient experienced a 20-fold increase in ALT and AST (1010/927 U/L) and needed hospital readmission. The CD4+ T-cell count and CD4/CD8 ratio were increased to 100/µL (14%) and 0.23, respectively (Figure 1 and Table 1).

Other viral causes of acute hepatitis were ruled out: hepatitis E virus (HEV)–RNA and hepatitis delta virus (HDV)–RNA were undetectable in plasma; hepatitis A virus (HAV)–immunoglobulin (Ig)M was absent in plasma (while HAV-IgG was detected, indicating previous HAV infection); CMV-DNA was no longer detectable in plasma at the time of hepatic flare; and Epstein-Barr virus (EBV)–DNA was detected in plasma below the limit of quantification (55 copies/mL).

Autoantibodies (antinuclear antibodies [ANA], smooth muscle antibodies [SMA], anticentromere antibodies, anti-double-strand DNA [dsDNA] antibodies, antibodies to extractable nuclear antigens [ENA], p- and c-antineutrophil cytoplasmic antibodies [ANCA]) were all negative. Moreover, total IgA, IgG, and IgM were within the laboratory reference values. Considering the patient's age, the absence of autoantibodies, the normal levels of IgG, and the evidence of active HBV replication, the diagnosis of autoimmune hepatitis was considered unlikely [15].

A liver biopsy was performed, and no histological signs of drug toxicity were identified. Suspecting an HBV IRIS flare, ART was continued, and ALT and AST progressively decreased during the following weeks. Noticeably, HBeAg seroconversion and anti-HBe antibody production (from HBeAg+/HBeAb– through HBeAg+/HBeAb+ to HBeAg–/HBeAb+) occurred during the ALT and AST flare observed 6–8 weeks after ART initiation (Figure 1 and Table 1). Moreover, HBsAg seroconversion was observed ∼88 weeks after ART initiation, characterized by HBsAg loss (as documented by both qualitative and quantitative HBsAg detection) and detectable anti-HBs antibodies, with a titer of 48.03 IU/L. The extension of the squamous cell carcinoma of the second finger of the left hand progressively reduced, concurrently with the CD4+ T-cell count recovery. Minor surgical resection of a limited part of the distal phalanx of the second finger was finally performed in association with local electrochemotherapy.

### Histology on Liver Biopsy

**Figure 2.**
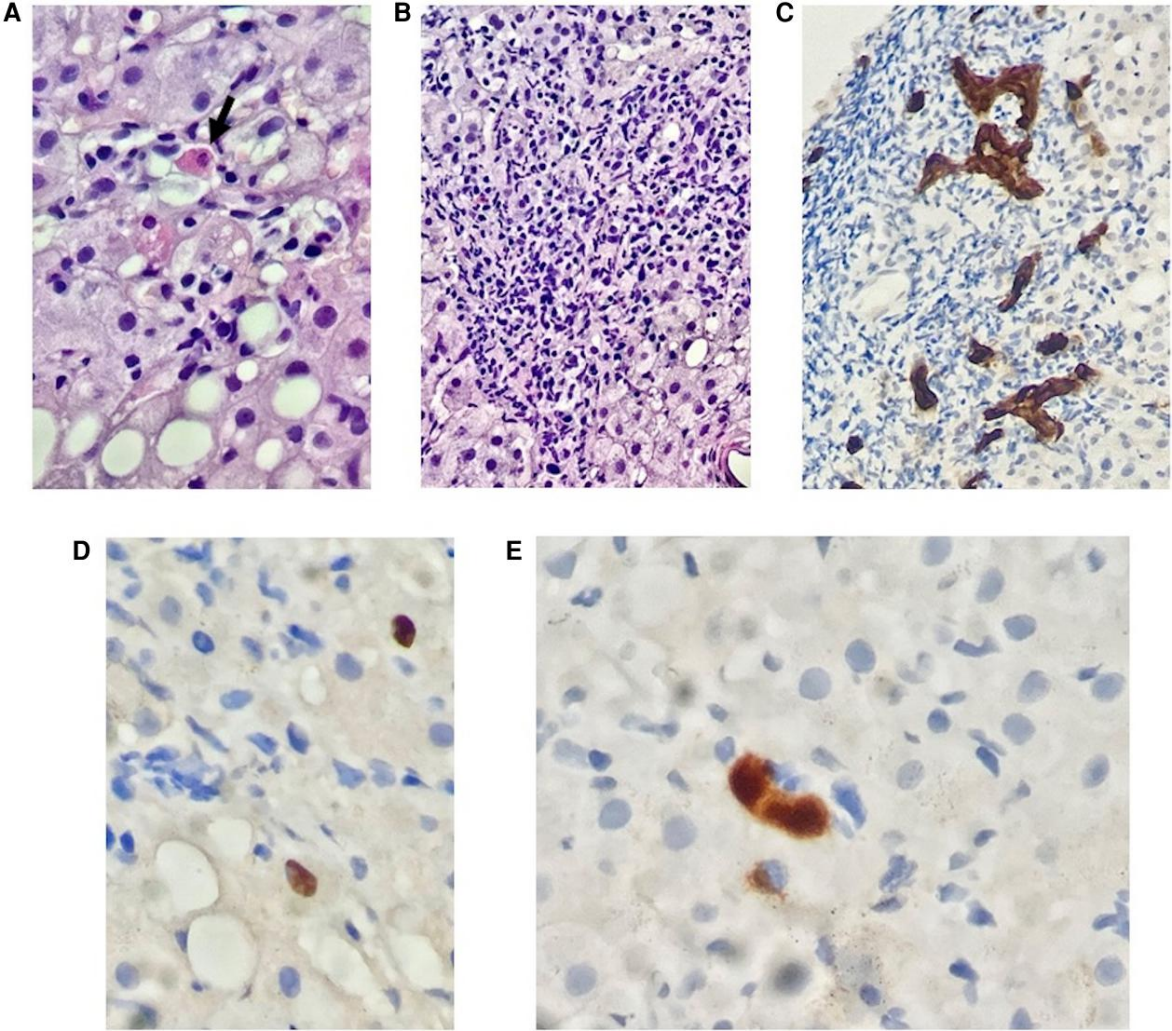
Liver biopsy. Hematoxylin and eosin staining of liver biopsy, 300X. Evidence of focal lobular necrosis with a Councilman's body (arrow), reactive atypical hepatocytes, macrovesicular steatosis, and focal cytoplasmic microvacuolation (A). Hematoxylin and eosin staining of liver biopsy, 120X. Portal space characterized by a chronic inflammatory cell infiltrate, composed predominantly of lymphomonocytes and rare eosinophils and plasma cells. Piecemeal necrosis is also evident (B). Immunohistochemical staining for CK7, 400X. Portal space with inflammation and irregular and abnormal neoproliferation of bile ducts (C). Immunohistochemical staining for HBcAg, 400X. Rare and scattered cells with nuclear positivity for HBcAg were observed (D). Immunohistochemical staining for HBsAg, 400X. Rare and scattered hepatocytes with positivity for HBsAg were observed (E). Abbreviations: CK7, Cutokeratin 7; HBcAg, HBV core antigen; HBsAg, HBV surface antigen; HBV, hepatitis B virus.

As reported, during the hepatic flare after ART initiation, a liver biopsy was performed. Histological examination showed chronic inflammation with piecemeal necrosis and lymphocyte infiltration of porto-biliary spaces (Ishak grading 9/18). Focal fibrosis was identified (Ishak staging 4–5/6). No evidence of drug toxicity was detected. Immunohistochemical staining for HBV core antigen (HBcAg) showed multiple focal nuclear staining of hepatocytes; rare HBsAg staining of hepatocytes was also detectable (Figure 2). No specific staining for CMV was performed on liver tissue. However, cytomegalic inclusion bodies were not detected in routine hematoxylin and eosin–stained sections of the liver biopsy. Moreover, at the time of the hepatic flare, plasma CMV-DNA was undetectable.

### Virological Characterization in Peripheral and Liver Compartments

In sequencing the genomic region encoding RT/HBsAg, no drug resistance mutations in RT were found; conversely, the HBsAg mutations Y100C, R122K, and T131N, associated with immune escape, were identified [16, 17].

**Figure 3.**
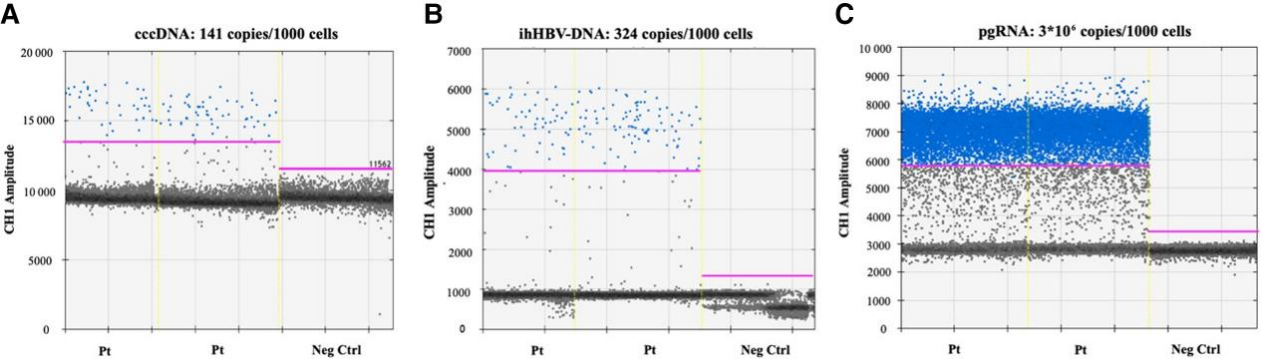
Intrahepatic HBV markers. The figure shows the plots obtained from ddPCR quantification of circular covalently closed DNA in duplicate (A); total intrahepatic HBV DNA in duplicate (B); and HBV pregenomic RNA in duplicate (C). Negative controls are also represented. The vertical axis represents the amplitude of the signals obtained by amplification. For each quantification, a positive threshold (magenta horizontal solid line) was set up, considering the highest amplitude of negative controls. The row quantification obtained by ddPCR was normalized for the volume of amplified DNA or RNA, the extraction volume, and the number of cells to obtain the correct number of copies in the original sample. Abbreviations: cccDNA, circular covalently closed DNA; ddPCR, droplet digital polymerase chain reaction; HBV, hepatitis B virus; ih, intrahepatic; Neg Ctrl, negative control; pgRNA, pregenomic RNA; Pt, patient.

Molecular investigations performed on the liver biopsy revealed a substantial burden of HBV intrahepatic (ih) reservoir, with ihHBV-DNA of 324 copies/1000 cells and cccDNA of 141 copies/1000 cells, coupled with a massive cccDNA transcriptional activity, as attested by a pgRNA level of 3.3 × 10^6^ copies/1000 cells (Figure 3 and Table 1).

### Immunological Characterization

**Figure 4.**
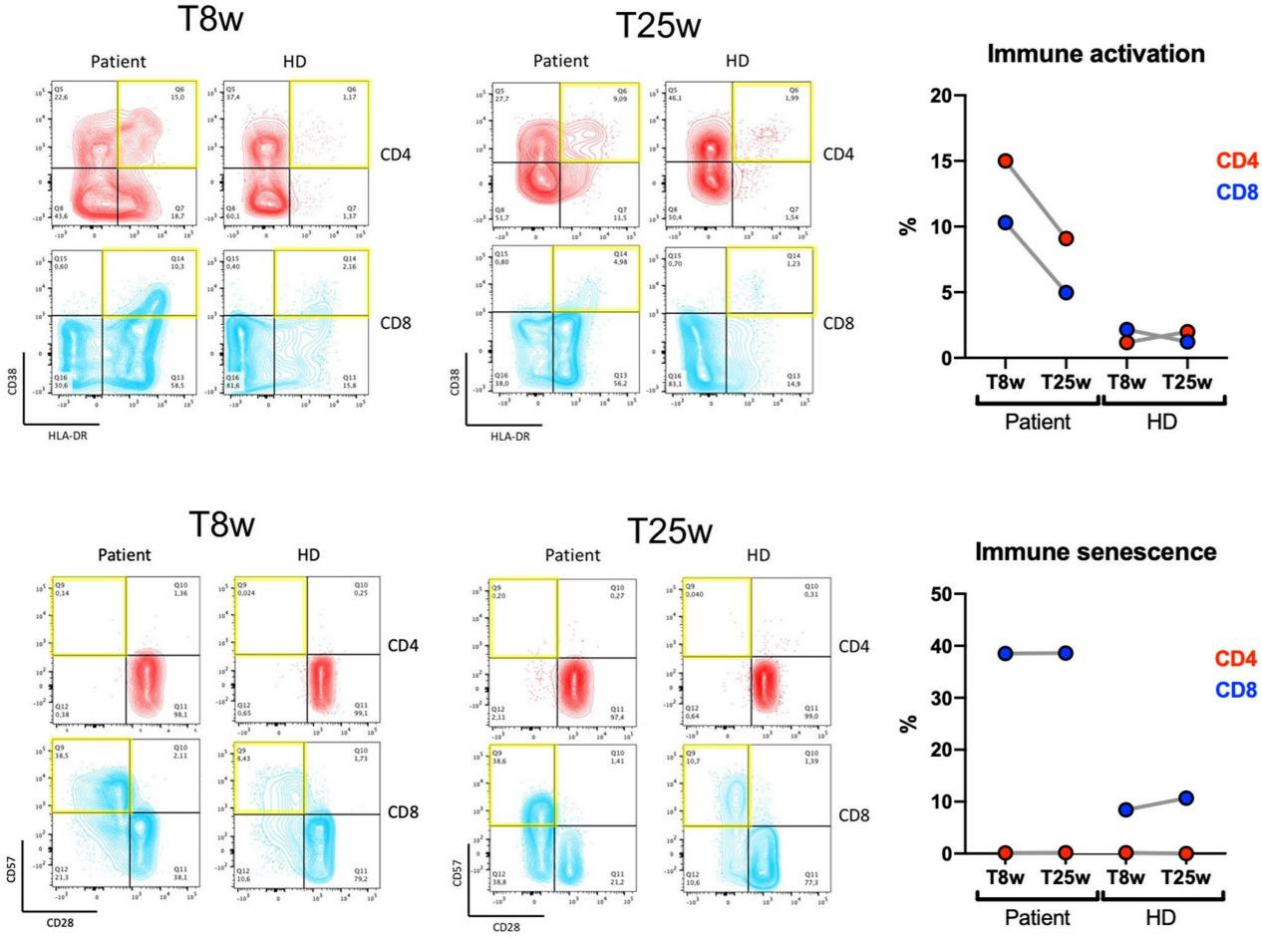
T-lymphocyte immune activation and senescence. CD4+ and CD8+ T-lymphocyte characterization at 8 weeks (T8w, during the hepatic flare) and 25 weeks (T25w, at liver enzyme normalization) after ART initiation. A, Immune activation was assessed by considering double-positive CD38 and HLA-DR CD3+ CD4+ and CD3+ CD8+ events. B, Immune senescence was assessed by considering CD28-negative CD57-positive CD3+ CD4+ and CD3+ CD8+ events. For each phenotypic characterization, an age- and sex-matched healthy donor was analyzed as a reference. The yellow squares highlight the populations of interest. Abbreviation: HD, healthy donor.

Peripheral blood T-lymphocyte phenotyping was performed 8 weeks after ART initiation, during the 20-fold liver enzyme elevation (T8w), and at 25 weeks after ART initiation (T25w), when liver enzymes had returned to a normal range. The study of T-cell activation, defined as the coexpression of CD38 and HLA-DR on either CD3+ CD4+ or CD3+ CD8+ cells, showed an elevated percentage of immune-activated T cells at T8w, with a considerable reduction at T25w. Conversely, immunosenescent T-cell percentages, defined as CD28-negative CD57-positive CD3+ CD4+ and CD3+ CD8+ cells, remained unchanged at the 2 time points (Figure 4). For both time points, peripheral blood T-lymphocyte phenotyping of an age- and sex-matched healthy donor was performed in parallel as a reference.

## DISCUSSION

In this clinical case, we report a hepatic flare in a patient with a newly diagnosed HIV and HBV coinfection 6 weeks after BIC/FTC/TAF initiation. The episode was self-limiting and resolved without ART discontinuation and led to the achievement of HBsAg seroconversion (HBsAg-/HBsAb+) with no detection of serum HBV-DNA.

Hepatic flare after ART initiation has been reported in 2%–14% of PWH, and the risk significantly increases in HBV- or HCV-coinfected subjects [9, 18]. In addition to drug-induced toxicity and direct viral damage, immune restoration, particularly a few weeks after ART initiation, can contribute to the development of hepatic flares in HIV/HBV-coinfected subjects. IRIS represents an inflammatory condition associated with paradoxical worsening of a preexisting infection, either previously recognized or occult [10]. In PWH, IRIS is usually associated with (i) low baseline CD4+ T-cell count, (ii) positive virologic and immunological response to ART, (iii) clinical manifestations of an inflammatory condition, (iv) temporal association between IRIS onset and ART initiation (2–8 weeks), and (v) absence of other alternative conditions, such as drug side effects, ineffective ART, poor compliance, or other concomitant infections [19]. In our case, all the abovementioned conditions were met.

In a randomized clinical trial including 36 HIV/HBV-coinfected subjects in Thailand, a hepatic flare within 12 weeks of ART initiation (including lamivudine and/or tenofovir) was observed in 22% of patients (median time to liver enzyme peak of 56 days). Seventy-five percent of patients experienced HBeAg loss, 5.6% had HBsAg loss, and 2.7% also showed HBsAg/HBsAb seroconversion during the 48-week observation period [20]. Accordingly, in our case, HBeAg/HBeAb seroconversion occurred 6–8 weeks after ART initiation, while HBsAg/HBsAb seroconversion was observed 88 weeks after ART initiation. A hepatic flare after ART initiation in HIV/HBV-coinfected subjects can be interpreted as IRIS, particularly when a subsequent rapid decrease in HBeAg and HBsAg levels and eventually seroconversion are observed. In this context, ART continuation with close monitoring of liver function tests is recommended [21].

Few studies have addressed the issue of HBV-related IRIS characterization in liver biopsies in PWH [22, 23]. In our case, histological findings excluded drug-related toxicities but showed liver inflammation with lymphocyte infiltration and HBcAg nuclear staining in the hepatocytes, consistent with the description reported by Rowley and colleagues [23]. Molecular biology analyses performed on liver tissue supported the existence of a substantial HBV intrahepatic reservoir characterized by extremely high transcriptional activity, as attested by the detection of high levels of HBV pgRNA. This elevated HBV-replicative activity coupled with massive viral antigen production might have offered good grounds for eliciting the occurrence of IRIS.

During HBV-related IRIS, hepatic damage is secondary to the recruitment of both antigen-specific lymphocytes and non-antigen-specific mononuclear cells to the liver and is mediated by CD8+ T-cell cytotoxicity and cytokine production [8, 10]. IRIS is also characterized by systemic inflammation, with detectable immune activation markers in the peripheral blood [8]. In our case, CD4+ and CD8+ T-cell immune activation was increased 8 weeks after ART initiation, concomitantly with hepatic flare, and substantially decreased after liver enzyme normalization 25 weeks after ART initiation, although it remained altered compared with a healthy donor. Conversely, immune senescence levels remained unchanged over time. Notably, during the 88-week follow-up period, the patient showed a remarkable increase in CD8+ T-lymphocytes, with persistence of a low CD4/CD8 ratio. This phenomenon has already been observed in PWH with HBV coinfection, lacking HBsAb production, and with AIDS events at HIV diagnosis [6].

It is significant to mention that, in this clinical case, the hyperactivation of the immune system played a pivotal role in promoting the control of HBV replication, thus triggering the achievement of HBsAg seroconversion during treatment with TAF/FTC. This is in line with a recent study showing that the hepatic flare caused by IRIS is a factor correlated with HBsAg loss in HIV/HBV-coinfected patients (HBsAg loss observed in 73.3% of patients with vs 20.9% of patients without IRIS-driven hepatic flares) [24]. Overall, this also reinforces the role of immunomodulant agents in supporting strategies aimed at achieving HBV functional cure.

In this case, the HBV clinical strain harbored 3 amino acid substitutions in the major hydrophilic region of HBsAg, which spans from amino acids 99 to 169 and includes the most antigenic determinant of the S gene (“a” determinant, amino acids 120–147) [16]. Nevertheless, the immune system was able to limit viral replication, reduce HBsAg production, and achieve HBsAg/HBsAb seroconversion 2 years after ART initiation.

Although CMV-DNA was undetectable in plasma during the hepatic flare, one limit of the study could be represented by the lack of CMV-specific staining on liver biopsy to definitely rule out CMV contribution to the hepatic damage.

At week 135 from ART initiation HIV-RNA was still undetectable, HBV-DNA was detectable below the quantification limit (<10 IU/mL), quantitative HBsAg was 0.00 IU/mL, and HBsAb titer rose to 3247 IU/mL. Liver enzymes were within the laboratory reference values, and the CD4+ T-cell absolute count was 352 cells/µL with a CD4/CD8 ratio of 0.19.

A further longitudinal evaluation is necessary to verify the maintenance of HBsAg loss over time in case of a potential subsequent simplification to a TDF/TAF-sparing antiretroviral treatment. Indeed, previous studies have shown that the withdrawal of HBV active drugs can predispose patients to reactivation of HBV replication in HBcAb+/HBsAg- PWH, supporting the importance of establishing proper monitoring of these patients [25, 26].

With this case, we describe a multidimensional approach for the characterization of HBV-related IRIS, in which histological, molecular biology, and immunological investigations were performed.
